# Focused ultrasound using a novel targeting method four-tract tractography for magnetic resonance–guided high-intensity focused ultrasound targeting

**DOI:** 10.1093/braincomms/fcac273

**Published:** 2022-10-25

**Authors:** Fabricio S Feltrin, Rajiv Chopra, Nader Pouratian, Mazen Elkurd, Rasheda El-Nazer, Lauren Lanford, William Dauer, Bhavya R Shah

**Affiliations:** Focused Ultrasound Lab and Program, Department of Radiology, UTSW Medical Center, Dallas, TX 75235, USA; Focused Ultrasound Lab and Program, Department of Radiology, UTSW Medical Center, Dallas, TX 75235, USA; Department of Neurological Surgery, UTSW Medical Center, Dallas, TX 75235, USA; O’Donnell Brain Institute, UTSW Medical Center, Dallas, TX 75235, USA; O’Donnell Brain Institute, UTSW Medical Center, Dallas, TX 75235, USA; Department of Neurology, UTSW Medical Center, Dallas, TX 75235, USA; O’Donnell Brain Institute, UTSW Medical Center, Dallas, TX 75235, USA; Department of Neurology, UTSW Medical Center, Dallas, TX 75235, USA; Focused Ultrasound Lab and Program, Department of Radiology, UTSW Medical Center, Dallas, TX 75235, USA; O’Donnell Brain Institute, UTSW Medical Center, Dallas, TX 75235, USA; Department of Neurology, UTSW Medical Center, Dallas, TX 75235, USA; Focused Ultrasound Lab and Program, Department of Radiology, UTSW Medical Center, Dallas, TX 75235, USA; Department of Neurological Surgery, UTSW Medical Center, Dallas, TX 75235, USA; O’Donnell Brain Institute, UTSW Medical Center, Dallas, TX 75235, USA; Advanced Imaging Research Center, UTSW Medical Center, Dallas, TX 75235, USA; Center for Alzheimer’s and Neurodegenerative Diseases, UTSW Medical Center, Dallas, TX 75235, USA

**Keywords:** focused ultrasound, FUS, HIFU, essential tremor, diffusion tensor imaging

## Abstract

Magnetic resonance–guided high-intensity focused ultrasound thalamotomy is a Food and Drug Administration–approved treatment for essential tremor. The target, the ventral intermediate nucleus of the thalamus, is not visualized on standard, anatomic MRI sequences. Several recent reports have used diffusion tensor imaging to target the dentato-rubro-thalamic-tract. There is considerable variability in fibre tracking algorithms and what fibres are tracked. Targeting discrete white matter tracts with magnetic resonance–guided high-intensity focused ultrasound is an emerging precision medicine technique that has the promise to improve patient outcomes and reduce treatment times. We provide a technical overview and clinical benefits of our novel, easily implemented advanced tractography method: four-tract tractography. Our method is novel because it targets both the decussating and non-decussating dentato-rubro-thalamic-tracts while avoiding the medial lemniscus and corticospinal tracts. Our method utilizes Food and Drug Administration-approved software and is easily implementable into existing workflows. Initial experience using this approach suggests that it improves patient outcomes by reducing the incidence of adverse effects.

## Teaser

Feltrin et al provide a technical note and clinical results from a novel targeting method for magnetic resonance guided high intensity focused ultrasound (MRgHIFU ) thalamotomy, 4 tract tractography. This approach improves the adverse effect profile of MRgHIFU while maintaining a sustained tremor response.

## Graphical Abstract

**Graphical abstract:**
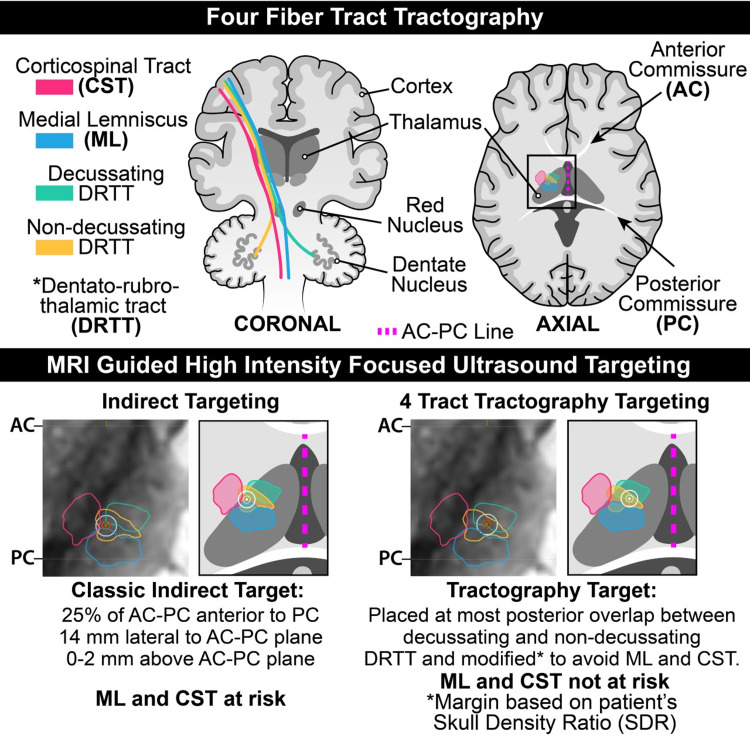

## Introduction

Magnetic resonance–guided high-intensity focused ultrasound (MRgHIFU) ablation of the ventral intermediate (VIM) nucleus of the thalamus is an incision-less, Food and Drug Administration (FDA) –approved procedure for essential tremor (ET) and Parkinson’s disease tremor. The efficacy and safety of MRgHIFU depend upon precise targeting of subcortical targets. Standard imaging methods are unable to delineate the VIM, necessitating landmark-based (indirect) targeting approaches.^1^ Indirect targeting is a workhorse of functional neurosurgery, but limitations of indirect targeting include individual variations in anatomy and function, subjectivity in selecting anatomic landmarks and targets and the effects of underlying pathological derangement on intracranial structures.^2,3^ Although indirect targeting can identify a subcortical region in the vicinity of the VIM, a lack of well-defined boundaries limits accurate identification in high-resolution MRI. During deep brain stimulation (DBS), microelectrode recordings (MERs) can be used to delineate the VIM. However, since MRgHIFU is an incisionless procedure, MER recordings cannot be obtained.^4^ The pivotal multicenter, randomized clinical trial studying MRgHIFU for ET used indirect targeting.^5^ Thirty-eight percent of subjects developed numbness and paraesthesia, 20% developed objective ataxia and 4% developed weakness. At 1 month, 29% of patients still had numbness and paraesthesia, 11% still had subjective ataxia and 4% still had weakness. At 3 months, numbness and paraesthesia decreased to 25% and objective ataxia to 3.6%, but weakness was unchanged. Similarly, in the most recent and largest retrospective study by Lak *et al.* side effects at 1 month included gait imbalance (46%), motor weakness (15%) and sensory deficits (33%).^6,7^ At 3 months, gait imbalance persisted in 26%, motor weakness in 7% and sensory deficits in 25%. This study also utilized indirect targeting but modified the indirect target to treat at 2 mm above AC-PC. It is well established that adverse effects remain a primary limitation of MRgHIFU and that the incidence of adverse effects is greatest during the first month after treatment.^5,7^ While oedema may account for transient side effects, limitations of indirect targeting may contribute to side effects still present after 3 months.

Advanced MRI techniques such as diffusion tensor imaging (DTI), quantitative susceptibility mapping (QSM) and fast grey matter inversion recovery (FGATIR) can provide imaging surrogates for underlying anatomy. FGATIR and susceptibility weighted imaging have been previously used to improve the targeting of the globus pallidus internus (GPi) and subthalamic nucleus (STN), but no such imaging modality exists for the VIM. DTI is the most promising of these advanced imaging techniques because it can identify white matter tracts to target as well as ones to avoid.^3,8,9^ The dentato–rubro–thalamic-tract (DRTT) represents an important target for tremor control. The DRTT are a pair of white matter tracts that run from the ipsilateral (non-decussating DRTT) and contralateral (decussating DRTT) dentate nuclei and connect the ipsilateral red nucleus, thalamus and motor cortex. The DRTT has been previously validated as a target by several DBS studies.^10,11^ The proximity of the DBS leads to the DRTT, which correlates positively with treatment response in ET.^12^ A prospective clinical trial using tractography to target the DRTT with MRgHIFU eliminated post-treatment weakness and numbness.^13^ However, transient subjective post-treatment imbalance was reported in 30% of the patients but resolved by 12 months.^13^

**Figure 1:**
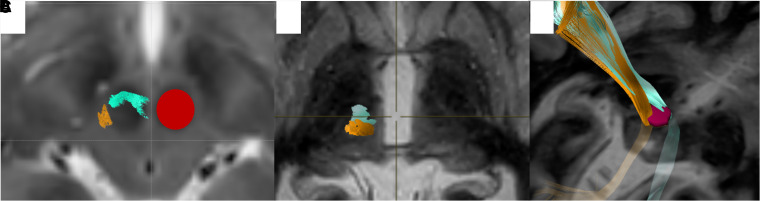
**The dDRTT and ndDRTT projected onto a FGATIR image.** (**A**) At the level of the red, the dDRTT (green) is larger and travels along the anterior margin of the red nucleus; the ndDRTT (gold) is smaller and at the posterior margin of the red nucleus (underlying red nucleus depicted on T2 weighted image, contralateral red nucleus ROI in red). (**B**)At the level of the AC-PC, the dDRTT remains anterior to the ndDRTT (**C**) 3D representation of the tracts.

Although DTI has been shown to be clinically useful, a lack of standardization and technical challenges in acquiring high-fidelity tractography has limited widespread adoption.^8^ There are two main approaches to fibre tracking. Although probabilistic tractography can account for crossing fibres and is more accurate, only deterministic tractography can be processed in FDA-approved software.^14–16^ While image distortion correction and eddy current correction can improve the accuracy of DTI,^17^ an understanding of anatomy is critical to accurate targeting.^18^ For example, previously published methods for DTI-based targeting attempt to identify some combination of three white matter tracts: corticospinal tract (CST) connecting primary motor cortex (PMC) to the spinal motor neurons, medial lemniscus (ML) connecting primary sensory cortex to trigeminal and spinal sensory neurons and the non-decussating component of the dentate-rubro-thalamic-tract (ndDRTT). These methods do not fully consider the implications of the underlying anatomy. For example, detailed anatomic studies have shown that there are two distinct DRT tracts, the ndDRTT and the decussating component DRTT (dDRTT). The dDRTT is larger and courses anterior to the red nucleus (Figs. 1A and C) and remains more anterior to the ndDRTT at the level of the AC-PC plane (Figs. 1B and C).

The dDRTT bifurcates into two fibre bundles which insert into the VIM and the VOp. The ndDRTT courses posterior to the red nucleus and lies immediately anterior to the ML (Fig. 1A). The ndDRTT inserts into the VIM. Above the level of the thalamus, both DRTT components connect to the motor cortex.^19,20^ The importance of identifying the relative position of these two tracts is underscored by studies that have shown that the proximity of the DBS leads to both the dDRTT and ndDRTT, which positively correlates with tremor improvement.^21^ These observations suggest that stimulation of both components of the DRTT plays a key role in tremor suppression. This view is supported by evidence that thalamic connectivity to the premotor and supplementary motor cortices plays an even more critical role in tremor suppression than connectivity to the motor cortex.^9,21^ To incorporate this anatomic and functional knowledge into our treatment algorithm, we developed a novel, clinically feasible, standardized tractography methodology for HIFU targeting that uses BrainLab Elements (BrainLab, Munich, Germany). BrainLab is a widely used, FDA-approved, neurosurgical planning software. This manuscript is a brief technical report on our methodology as well as a report on 3-month outcomes in 18 consecutively treated ET patients. The definition of ET followed the consensus statement on the classification of tremors from the task force on tremors of the International Parkinson and Movement Disorder Society.^22^

## Materials and methods

### Patient selection

The data presented in this manuscript reflect 18 consecutive ET patients (M = 10; F = 8) who underwent MRgHIFU from March 2021 to October 2021. Patients had at least 3 months of follow up for tremor and adverse effect assessment by a movement disorder neurologist.

### Clinical assessment

A movement disorder neurologist is part of our treatment team and performs neurologic assessments before, during and after the treatment. Assessments include a cranial nerve exam, motor strength, coordination evaluation, gait testing, superficial and deep sensitivity in all extremities and face, tremor assessment with finger to nose testing, hands positioned at near and far, spirals and axial tremor assessment, including voice tremor. Immediately after and 2 days after the procedure, each patient is also evaluated by a movement disorder trained physician assistant and neuroradiologist. The neurologic assessment consists of a cranial nerve exam, speech evaluation, sensory and motor strength testing, tremor exam as described above as well as a gait exam. The gait assessment evaluates ataxia with tandem walking and stance in stationary and tandem positions. Truncal ataxia is further assessed by having patients sit with arms outstretched and eyes closed without foot support.

### Spiral assessment

Two movement disorder neurologists and one movement disorder trained PA independently and blindly rated spirals using the modified Washington Heights-Inwood Genetic Study of Essential Tremor (WHIGET)^23^ tremor rating scale before and after MRgHIFU at 1 and 3 months after MRgHIFU. The PA was trained by our movement disorder neurologists to independently evaluate tremor in a clinic, with 8 years of experience in both clinical and research environments. The agreement between raters was quantified with a kappa value of 0.95. A blinded consensus conference was held to finalize the scores. Examples of spirals and scores are included in the Supplementary Fig. 1. The total improvement in tremor score was calculated as follows:

$$\mathrm{Tremor}\;\;\mathrm{Improvement}=\frac{\mathrm{Pre}\;\mathrm{Treatment}\;\mathrm{Score}\text{-}\mathrm{Post}\;\mathrm{Treatment}\;\mathrm{Score}}{\mathrm{Pre}\;\mathrm{Treatment}\;\mathrm{Score}}$$

### MRI acquisition

A 60-minute MRI scan with DTI is performed on a Phillips 3T MR Scanner (Philips, Best, the Netherlands). The sequences include isotropic T2W 3D TSE (FOV 24 cm, matrix 268 × 268 mm, TR = 2500, TE = 255.56 thickness 0.9 mm, GAP = 0 mm, spacing = 0.9 mm); FGATIR (FOV 25 cm, Matrix 256 × 256 TR = 6.615, TE = 2.949, thickness = 0.9 mm, GAP = 0, spacing = 0.9 mm); axial 3DT1 TFE (FOV = 24 cm, matrix 268 × 187, TR = 8588 ms, TE = 3.93 ms, GAP = 0, spacing = 0.9 mm); 32 direction DTI (FOV 24 × 24 × 15 cm, matrix 96 × 96, *B* value = 800, TR = 3400 TE = 84.5, acquisition voxel = 2.5 mm, thickness 2.5 mm, spacing 2.5 mm, GAP = 0, SNR = 0.99, slices = 60, Halfscan factor = 0.84).

### DTI registration with structural images and distortion correction

The images are uploaded to BrainLab. The DTI images are rigidly co-registered to both structural FGATIR and 3D TSE T2W sequences using image fusion, both with a 0.7 mm isotropic resolution. Co-registration and distortion correction of DTI and anatomical images is based on inverse contrast normalization, which is performed in Brainlab elements.^24^ A corrected DTI image set is generated and utilized for fibre tracking.

### Fibre tracking

**Figure 2:**
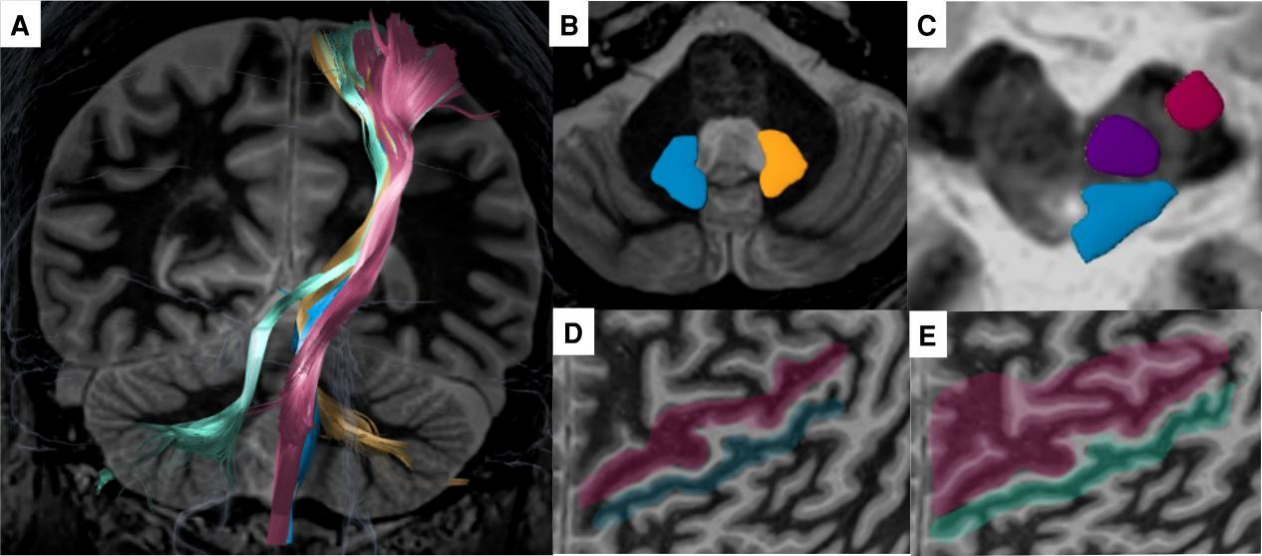
**Four tracts and ROIs are utilized for tracking**. (**A**) Four-tracts are projected over a coronal FGATIR image. The ROIs are drawn **over** axial FGATIR images (**B–E**). (**B**) Left dentate nucleus (gold) and right dentate nucleus (light blue). (**C**) Left cerebral peduncle ROI for CST (red), left red nucleus (purple) and medial lemniscus (light blue) are drawn over the midbrain. (**D**) ROIs for PMC (pink) and primary sensory cortex (green) on a flattened projection. (**E**) One ROI for primary, supplementary and premotor cortices (pink) for the dDRTT.

**Figure 3:**
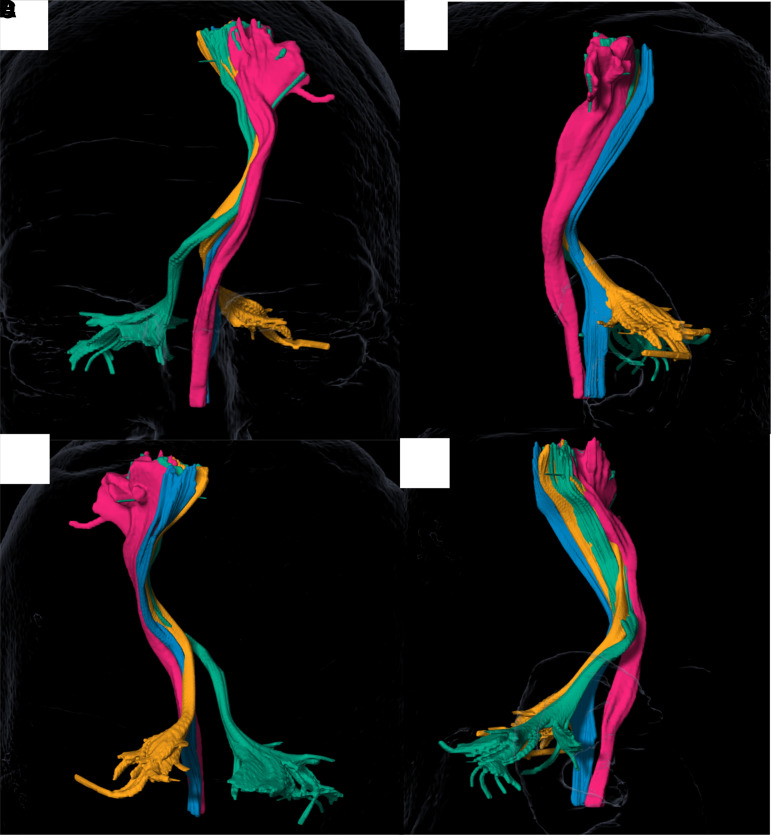
**3D rendering of four tracts in multiple projections**. (**A**) Anterior, (**B**) left lateral, (**C**) posterior, (**D**) right lateral.

**Figure 4:**
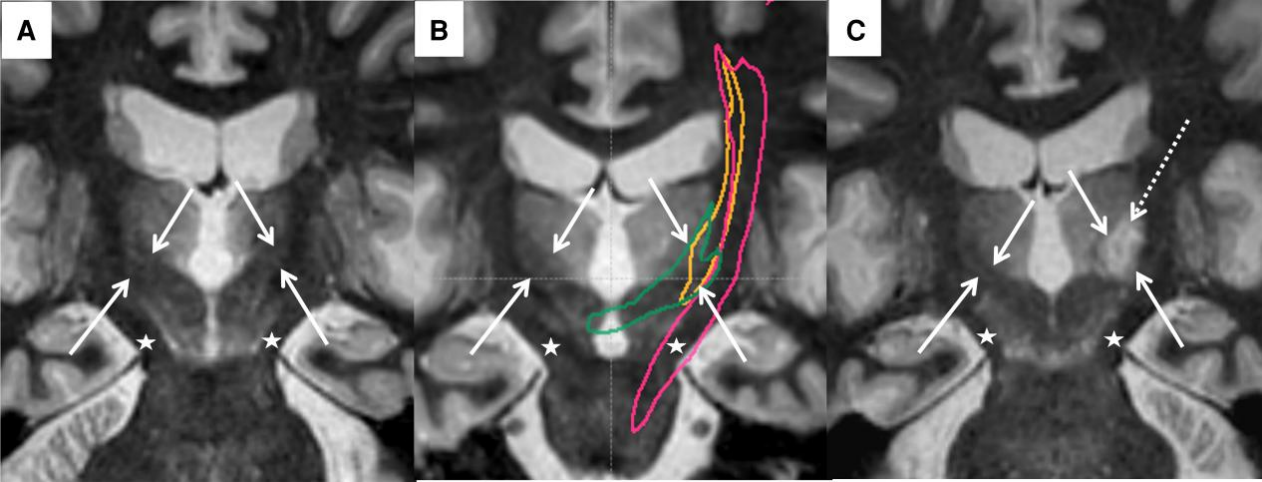
**Coronal oblique FGATIR images before and 1-month after treatment, demonstrating the relationship of the white matter tracts to the ablation zone**. (**A**) Both components of the DRTT are demonstrated as a hypointense tract extending from the red nucleus to the thalamus (white arrows). The CST is demonstrated as a hypointense tract extending from the cerebral peduncle to the thalamus (star). (**B**) The ndDRTT (gold), dDRTT (green) can be seen in the expected location of the DRTT (arrows) and CST (pink) also in its expected location (star). (**C)** The treatment ablation zone (white dotted arrow) matches the position of the DRTT as seen in pre-treatment FGATIR and tractography (**A** and **B**).

Fibre Tracking in BrainLab is a deterministic tensor deflection streamline approach. The user can enter manually drawn regions of interest (ROIs) as seed points for the algorithm. For the CST, ROIs are drawn in the PMC at the precentral gyrus and ipsilateral cerebral peduncle. For the ML, ROIs are drawn in the postcentral gyrus and dorso-lateral aspect of the midbrain, extending from the quadrigeminal plate anteriorly, just below the level of the red nucleus. For the ndDRTT, ROIs are drawn in the PMC (M1), ipsilateral red nucleus and ipsilateral dentate nucleus (Fig. 2). For the dDRTT, ROI are drawn in the PMC (M1), ipsilateral red nucleus and contralateral dentate nucleus. The ROIs are drawn using both the FGATIR and T2 images. The red nucleus is better delineated on the T2 images and thus we use this sequence for drawing the red nucleus ROI. Although BrainLab offers automatic segmentation, we use manual segmentation. For all tracts, the same parameters are initially used fractional anisotropy (FA) = 0.2, minimum fibre length = 80 mm, maximum angle = 20°. Delineating the dDRTT has previously been reported to be challenging in commercially available software.^25^ When the dDRTT cannot be identified, our first step is to ensure that the red nucleus ROI is appropriate. Second, we modify fibre tracking parameters to match anatomic knowledge of fibre orientation and to follow previously published fractional anisotropy (FA) values (FA = 0.11, minimum fibre length = 40 mm, maximum angle = 50°).^26^ Third, a combined ROI that includes the PMC (M1), the supplementary motor cortex (SMC) and the premotor cortex (PreMC) can be substituted for the PMC (M1) ROI (Fig. 2). The anatomical boundaries of M1, SMC and PreMC of these areas have been previously defined and published (http://www.fmrib.ox.ac.uk/connect/definitions.html).^27^ In the cases presented in this manuscript, we only had to modify fibre tracking parameters once. 3D models of all four tracts are shown in Fig. 3. The resulting tracts are generated and overlaid on an FGATIR image (Fig. 4) to check for anatomical accuracy. We have consistently observed that we can identify the DRTT bundle (dDRTT and ndDRTT) (Fig. 4) on FGATIR images without tractography.^3^ While DTI is an echo planar imaging technique that requires large gradients that can result in artefacts, the FGATIR is a non-echo planar sequence that does not require post-processing. Although it is possible to identify the CST and directly target the DRTT bundle using FGATIR alone, it is not yet possible to delineate the contributions of the DRTTs or to identify the ML (to avoid it) during sonication.

### Generating a DICOM image with burned-in fibre tracts for treatment

**Figure 5:**
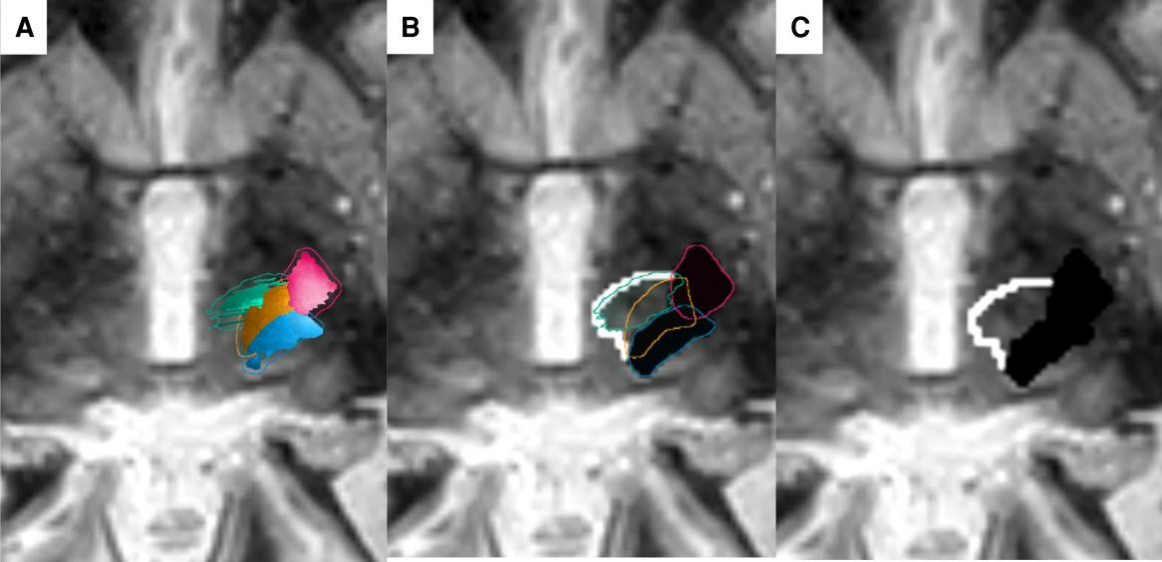
**Images demonstrating the process of combining tracts and burning them as objects for treatment**. (**A**) Demonstrates the dDRTT (green), ndDRTT (gold), CST (pink) and the ML (blue) on an axial FGATIR image. (**B**) Demonstrates the conversion of the CST and ML into a single black object and the dDRTT and nDRTT as a white contour on a FGATIR image. (**C**) The final image burned is a -in DICOM image and is utilized for treatment on a FGATIR image.

The BrainLab software does not allow for the tracts to be exported over the structural FGATIR or 3DT2W TSE images. For this reason, the tracts need to be converted into objects and burned into the structural images. The 3D object created by BrainLab is a representation of the voxels included in the tract streamline. Therefore, they are minimally larger than the original fibre bundles. The objects representing the fibre bundles are then burned into the FGATIR sequence. The final images are then uploaded to the ExAblate (ExAblate Neuro 4000, Insightec, Haifa, Israel) system. The burned-in image is created by first merging both DRTT components (decussating and non-decussating) into a single object with the ‘Union’ operation with the object manipulation tool in BrainLab. The combined dDRTT and ndDRTT now appear as a white outline on the T2W series. Next, the CST and ML are merged as another object and burned as a black object in the DICOM image created in the prior step (Fig. 5).

### Treatment planning

**Figure 6:**
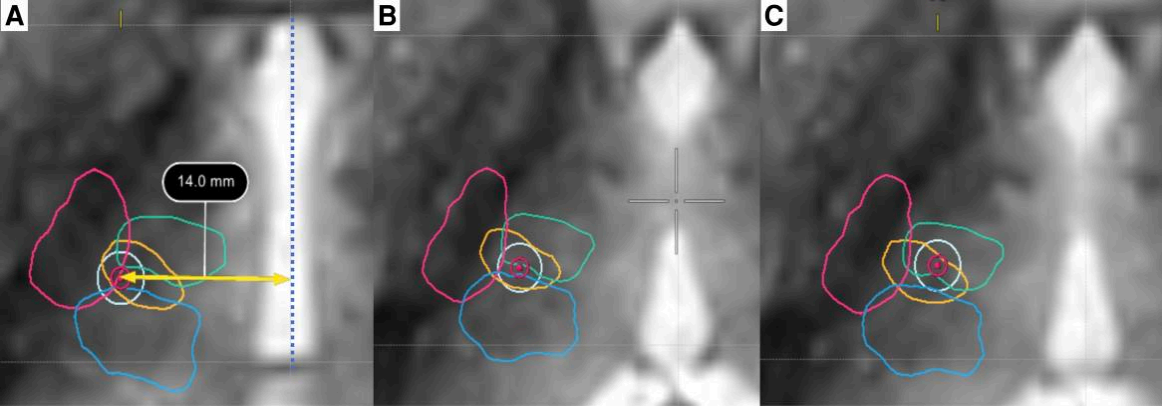
**Demonstration of the difference between indirect targeting and tractography-based targeting**. The ML (blue), CST (pink), dDRTT (green) and ndDRTT (gold) are demonstrated in images (**A–C**) in the axial plane. (**A**) Standard indirect target (14 mm lateral to AC-PC line and 10–11 mm lateral to 3rd ventricle wall) on overlaid on FGATIR showing the internal capsule, CST and ML are at risk. (**B**) The standard indirect target moved 2 mm superior to AC-PC. The target remains too lateral and posterior and overlaps with the ML and minimally with the internal capsule and CST. (**C**) Represents the final tractography-based target, which is centred at the most posterior junction of the dDRTT and ndDRTT and avoids the ML and CST.

Treatment planning is performed in trajectory planning in BrainLab. The AC and PC are manually selected, and the MRI data is reformatted in the AC-PC plane. Target coordinates are determined by using the standard indirect targeting method in relation to the AC-PC plane: 14 mm lateral to the mid-commissural point, 25% of the AC-PC length anterior to the PC and at the level of the AC-PC plane. Third, ventricle size is also accounted for using a modified indirect targeting methodology that estimates the target to be 10–11 mm lateral to the wall of the 3rd ventricle. The indirect coordinates are first modified to start 2 mm above the AC-PC plane instead of at the AC-PC plane. The rationale supporting this modification is that most HIFU lesions are ovoid in the craniocaudal direction. Starting above the AC-PC plane prevents inferior extension of the lesion. The first tractography-based target is then placed at the most posterior confluence of the dDRTT and ndDRTT. The ‘margin tool’ in BrainLab is used to identify potential overlap with the CST and ML in three planes. The final coordinate selection is modified to eliminate overlap (Fig. 6). Margin size varies by institution, some sites utilize margins up to 4 mm^10^. In our experience, an important consideration when determining the size of the margin is the skull density ratio (SDR). Patients with low SDRs often require longer sonication times at higher energies, which results in larger lesions with more oedema. In these patients, a larger margin (4 mm) between CST and ML may be important in reducing adverse effects. In patients with higher SDRs, the final lesion is typically a prolate spheroid that usually extends in the superomedial to inferolateral direction with a zone of restricted diffusion that measures 2–3 mm. It is important to note that the BrainLab burn-in process generates an approximately 0.5 mm margin surrounding the true white matter tract because it uses a voxel-wise approach. We routinely display the fibre bundle inside of the final burned-in tract to determine final margins. Finally, a second tractography-based target is placed at 4 mm above the AC-PC line at the most posterior confluence of the dDRTT and ndDRTT and the process is repeated. Since the shape and volume of the ablation are influenced by a subject’s SDR, deactivated ultrasound elements and other no-pass zones, tractography also enables a safe, real-time method to add additional targets in untreated tissue in safe regions during the treatment. This can improve permanence while avoiding adverse effects.

### Treatment

We follow a standard patient preparation protocol that has been previously described.^1^ The burned-in image is first registered to the head CT. Fiducials are placed at target locations as determined during treatment planning. ML and CST safety margins are verified. A real-time sagittal CISS sequence (FOV 32 cm, matrix 320 × 240 mm, TR = 8.64 TE = 4.32 thickness 1.5 mm GAP = 0.68 mm) is obtained. The AC and PC are identified and the images are reformatted into three planes. The midline is marked on a coronal image. The real-time MRI is registered to the treatment plan.

**Figure 7:**
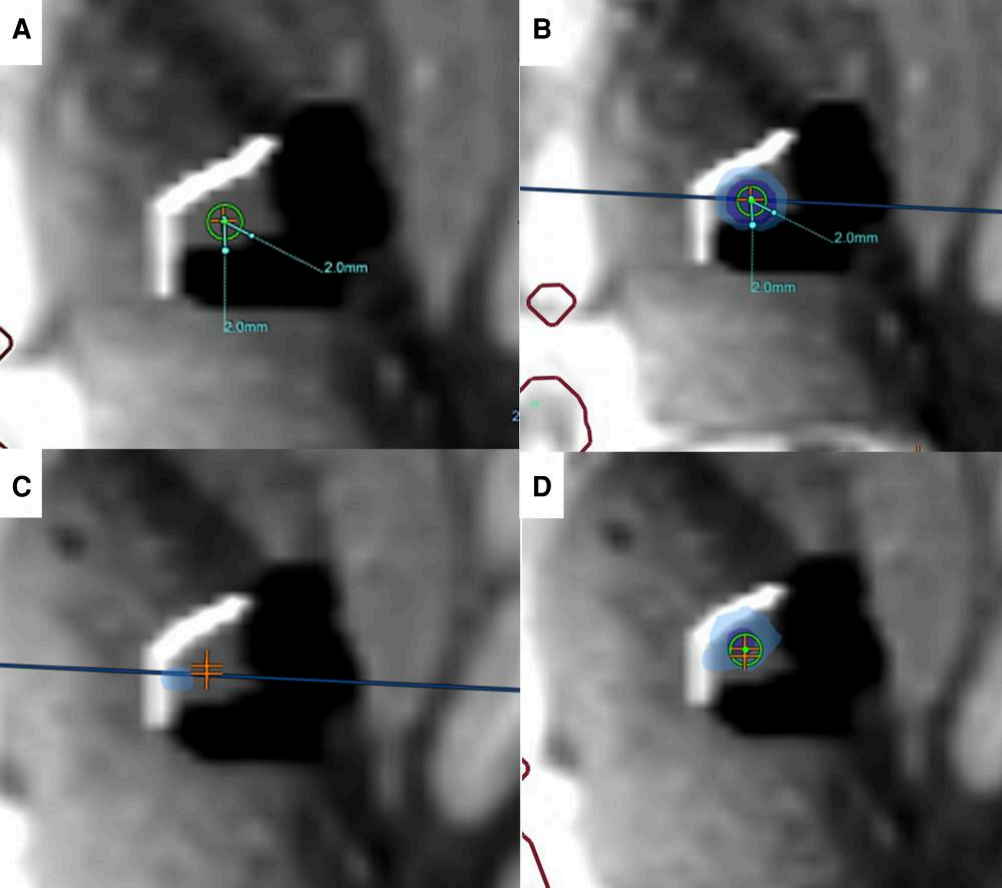
**Treatment images from the Insightec console**. (**A**) The first target (orange cross) is placed at the posterior confluence of dDRTT and ndDRTT with a 2 mm safety margin from ML/CST (black object). (**B**) After the sonication, the thermal dose is demonstrated in blue over the DRTT bundle. (**C**) A second target (orange cross) is placed superomedially within the DRTT bundle. (**D**). Thermal dose overlay after sonication at the second target. The dark blue represents an ablative thermal dose.

After each treatment sonication, the patient is evaluated for tremor response and side effects. Our desired thermal dose at each target is 56–57°C. The location of the second target can be modified (if necessary) in real-time using the thermal overlay function on tractography images. The final spot size and morphology are highly dependent on the SDR and location of deactivated elements. The ability to visualize the delivered thermal dose on tractography images can help identify untreated regions within the DRTT (Fig. 7). However, there are limitations to using the thermal dose maps. The thermal dose map is a 3D representation of a 2D measurement and is only accurate in the plane in which it was acquired. Nevertheless, it still provides an estimate of target coverage in real-time.

### Statistical analysis

Continuous variables were described as mean with standard deviations. Categorical variables were described as percentages. Agreement between observers was calculated with Cohen's Kappa test. The differences between the target coordinates and also the differences between the WHIGET scores were calculated using a paired *t*-test.

## Results

### Relationship of indirect target to tractography-based target

The indirect target was moved toall patients. The tractography based target was more anterior (range 0.1–1.9 mm; median = 0.65 mm) and more medial (range 0–2.3 mm; median—0.9 mm) to the indirect target (Table 1). The average Euclidean distance between the indirect target and the first tractography target was 1.80 mm ± 0.88 mm. There was a statistically significant difference between the indirect and tract-based coordinates for both anterior (*P* < 0.005) and lateral coordinates (*P* < 0.05). Immediate and one-month post-treatment MRI demonstrated no extension of the ablation zone to CST or ML. Treatment parameters are provided in Table 2.

**Table 1:** Comparison of indirect and tractography-based targets

Case	Indirect target	First tractography/sonication target coordinates	Second tractography/sonication target coordinates	Euclidian distance between indirect target and 1st sonication target (mm); assuming the indirect target is 2 mm above AC-PC
1	L = 14 mm A = 7.1 mm S = 0 mm	L = 13.8 mm A = 8.1 mm S = 2 mm	L = 14.6 mm A = 8.1 mm S = 3.3 mm	1.02
2	L = 14 mm A = 7.4 mm S = 0 mm	L = 13.2 mm A = 7.5 mm S = 2 mm	L = 13.2 mm A = 7.5 mm S = 4 mm	0.81
3	L = 14 mm A = 6.4 mm S = 0 mm	L = 13.8 mm A = 8.3 mm S = 3.5 mm	L = 15 mm A = 8.3 mm S = 5 mm	2.43
4	L = 14 mm A = 6.5 mm S = 0 mm	L = 13 mm A = 5.5 mm S = 2 mm	L = 14.5 mm A = 6.3 mm S = 5 mm	1.41
5	L = 14 mm A = 7.5 mm S = 0 mm	L = 13.9 mm A = 8 mm S = 2 mm	L = 16.2 mm A = 8.4 mm S = 4 mm	0.51
6	L = 14 mm A = 6.9 mm S = 0 mm	L = 11.7 mm A = 6.4 mm S = 2 mm	L = 15.1 mm A = P5.3 mm SI = 4mm	2.35
7	L = 14mm A = 6.4mm S = 0mm	L = 12.5mm A = 6.5mm S = 2mm	L = 12.5mm A = 4.5mm S = 4mm	1.50
8	L = 14 mm A = 7.4 mm S = 0 mm	L = 13.3 mm A = 7.6 mm S = 0.2 mm	L = 17.7 mm A = 9 mm S = 2 mm	1.94
9	L = 14 mm A = 7 mm S = 0 mm	L = 14.5 mm A = 8.0 mm S = 2.0 mm	L = 16 mm A = 8 mm S = 4 mm	1.12
10	L = 14 mm A = 7.6 mm S = 0 mm	L = 13 mm A = 8.3 mm S = 2.5 mm	L = 15.1 mm A = 8.5 mm S = 4.1mm	1.32
11	L = 14 mm A = 6.5 mm S = 0 mm	L = 11.9 mm A = 7.1 mm S = 2 mm	L = 12.8 mm A = 6.7 mm S = 2.9 mm	2.18
12	L = 14 mm A = 6.2 mm S = 0 mm	L = 9.7 mm A = 6.9 mm S = 1.4 mm		4.40
13	L = 14 mm A = 7 mm S = 0 mm	L = 13 mm A = 8 mm S = 2 mm	L = 14 mm A = 8.5 mm S = 4 mm	1.41
14	L = 14 mm A = 6.7 mm S = 0 mm	R = 12 mm A = 7.9 mm S = 2.0 mm	R = 12 mm A = 8.5 mm S = 4 mm	2.33
15	L = 14 mm A = 7.0 mm S = 0 mm	L = 12.4 mm A = 7.9 mm S = 1.5 mm	L = 13.3 mm A = 8 mm S = 2.5 mm	1.90
16	L = 14.5 mm A = 6.5 mm S = 0 mm	L = 14.4 mm A = 7.5 mm S = 2.0 mm	L = 15 mm A = 8 mm S = 4.1 mm	2.23
17	L = 14 mm A = 5.8 mm S = 0 mm	L = 14 mm A = 5.8 mm S = 2.0 mm	L = 15 mm A = 5.8 mm S = 4 mm	1.00
18	L = 14.2 mm A = 7.0 mm S = 0 mm	L = 13.8 mm A = 7.0 mm S = 2.0 mm	L = 13.8 mm A = 7.5 mm S = 4.4 mm	2.03
Average ± DP	L = 14.03 mm ± 0.12 A = 6.8 ± 0.49 mm S = 0mm	L = 13.0 ± 1.17 mm A = 7.4 ± 0.85 mm S = 2.0 ± 0.61 mm	L = 14.5 ± 1.45 mm A = 7.5 ± 1.16 mm S = 3.8 ± 0.78 mm	1.8 ± 0.88 mm

A, anterior; L, lateral; S, superior. Al measurements are bases in reference to AC-PC line.

**Table 2:** Treatment parameters summary

Case	SDR	Total sonications	Post-alignment sonication	Total elements	Skull surface	Total energy (J)	Maximum potency (W)	Maximum duration (s) of sonication	Maximum average temperature (°C)
1	0.60	10	4	973	378	45 150	621	12	58
2	0.66	10	6	937	323	53 075	847	13	56
3	0.59	9	5	949	329	43 303	659	16	59
4	0.56	11	5	961	405	111 115	866	26	57
5	0.47	11	4	920	340	50 934	624	17	62
6	0.45	13	5	935	355	85 534	1162	16	58
7	0.40	7	4	892	316	70 245	1051	23	56
8	0.45	10	6	758	305	51 113	682	18	59
9	0.49	9	6	817	364	89 791	1154	17	60
10	0.44	9	6	915	419	83 021	1053	17	56
11	0.45	8	5	763	279	35 473	831	13	56
12	0.38	6	2	822	275	106 800	1125	36	56
13	0.40	10	5	988	416	136 078	1056	31	57
14	0.55	11	5	889	331	62 262	1004	12	58
15	0.62	11	6	908	334	44 723	1013	10	56
16	0.40	12	7	947	340	84 200	1008	18	56
17	0.63	10	*6*	995	354	38 232	760	13	58
18	0.40	11	7	982	353	74 858	1041	16	57
Average ± DP	0.57 ± 0.1	9.8 ± 1.6	5.1 ± 1.1	908.3 ± 71.2	345.3 ± 39.9	70238.2 ± 27 562	919.8 ± 181.1	18 ± 6.9	57.7 ± 1.6

C, Celsius; J, Joules; W, Watts; s, seconds.

### Number of sonications

The total number of sonications ranged from 6 to 13, with an average of 9.9 ± 1.7. The number of post-alignment treatment sonications ranged from 4 to 7, with an average of 5.2 ± 1.2. One patient received 13 sonications, because of movement after four alignment sonications necessitating re-planning and the second round of alignment sonications. If removed from the analysis, the total number of sonications ranged from 6 to 12, with an average of 9.7 ± 1.6. Average SDR, average number of sonications and average thermal dose are provided in Supplementary Table 1.

### Target movements

In all 18 patients, a tremor response in the absence of adverse effects was seen at the initial tractography target. We consider the move from the first planned target to the second planned target as one target movement. Our total target movements ranged from 0 to 2 with an average of 1.1 ± 0.47. As described above, one patient moved after the alignment sonication and had to be re-planned. In another patient, we chose to ablate an additional target to improve axial tremor control.

### Clinical outcomes

**Figure 8:**
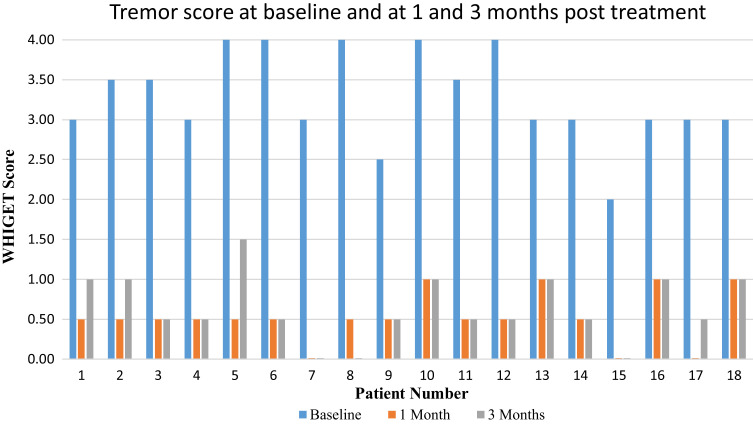
**The modified WHIGET spirals score is demonstrated for each time point for all 18 patients**. Baseline is exhibited in blue, 1 month after treatment in orange and 3 months after treatment in grey. Patients 7, 8 and 15 have maintained complete resolution of their tremors at 3 months.

Immediately following treatment, upper extremity tremors abated completely in all 18 patients. Using spirals as an objective measure of hand tremor score, we quantified an improvement of an overall average tremor score of 84% at 1 month and 81% at 3 months. There was a significant difference in the WHIGET scores at baseline versus 1 and 3 months (*P* < 0.001), but there was no difference in the score from 1 to 3 months (*P* = 0.2). Individual responses are shown in the bar graph (Fig. 8). No objective adverse side effects were detected during the treatments by a movement disorders neurologist. None of the subjects experienced weakness, numbness, paraesthesia, dysarthria or objective ataxia. Three subjects (∼16%) complained of subjective imbalance that was resolved in 1 month. Notably, 67% of subjects (*n* = 12) presented with voice and head tremors but subjectively reported axial and voice tremor abatement after treatment. This response persisted when seen in follow up neurology and head and neck clinics at 1 and 3 months. Because this was an unexpected outcome, direct quantitative assessments were not initially performed. We have subsequently incorporated pre- and post-procedure voice recordings and accelerometer measurements.

## Discussion

This manuscript provides a technical overview and describes several benefits of our novel, easily implemented advanced tractography method: four-tract tractography. Our method augments the existing body of tractography-based interventions by incorporating lessons learned from prior anatomical and functional studies. Although recent publications suggest that tractography-based methods are comparable to indirect targeting methods,^28^ > 10 years of multicenter DBS targeting demonstrate the benefits and feasibility of DTI targeting.^13,28^ Similar to previously published work, we demonstrate that the tractography-based target is generally more anterior and medial when compared with indirect coordinates.^13,29–31^ Notable exceptions include patients with an enlarged third ventricle. In these cases, the tractography-based target is more lateral than that predicted by the modified indirect targeting method (10–11 mm lateral to the wall of the third ventricle).

During MRgHIFU, the patient is evaluated for tremor response and for the development of adverse effects after each sonication. The most apparent benefit of tractography-based targeting is an immediate tremor response, decreasing or altogether obviating the need for target movement. Focused Ultrasound centres that rely on indirect targeting reposition the target an average of three times.^28^ Target repositioning is performed when there is an adverse effect or there is no tremor response at 52°. Target repositioning increases treatment time and can result in unintended sonication of the ML or CST. In patients with low SDR, the progressive loss of efficiency after repeated sonications may also preclude achieving an ablative thermal dose, resulting in a prolonged, more painful treatment or even a suboptimal treatment. We made an average of 1.1 target movements in these 18 patients. We counted moving from the initial target to the second target as one movement. One patient moved after the alignment sonications and we had to replan the procedure. Another patient had a prominent axial tremor and we treated an additional third target. We did not have to reposition the initial tractography-based target because of adverse effects or lack of tremor response.

As expected and concordant with previously published work using tractography for targeting^13^ we also demonstrate a decreased rate of sensory and motor adverse effects during and after treatment. Side effects such as weakness and sensory disturbances are associated with the extension of the ablation zone into the CST and ML, respectively.^32^ Direct visualization of these tracts allows the user to avoid them during treatment. Sonication of the DRTT can be associated with transient, subjective imbalance in the absence of objective ataxia.^32^ Therefore, the subjective transient unsteadiness experienced by patients is an outcome that they should be counselled about before the procedure. In our study, the only adverse effect was a transient imbalance in three subjects (16%) that resolved at 1 month. In the pivotal study,^5^ 29% of patients still had numbness and paresthaesia, 11% still had subjective ataxia and 4% had weakness at 1 month. Similarly, in the largest retrospective study evaluating MRgHIFU for ET, side effects at 1 month included gait imbalance (46%), motor weakness (15%) and sensory deficits (33%).^7^ When compared with the pivotal study^5^ in which indirect targeting was performed at the level of the AC-PC plane, more recent studies, including the largest retrospective HIFU study^7^ and our study, have shifted the technique to targeting 2 mm above the AC-PC plane. Although this is not routinely performed by all treatment centres, empirical evidence suggests it may be associated with safer outcomes and better tremor response. However, the incidence of adverse effects was similar between the pivotal trial^5^ and the study by Segar *et al*.^7^ Based on our experience, tractography helps avoid adverse effects from off-target sonication.

We also observed that our tractography method enables fewer sonications when compared with previously reported indirect targeting methods. However, the number of sonications is dependent on a multitude of other factors, for example, SDR. Therefore, although these differences cannot be directly and specifically attributed to differences in targeting methodology, we hypothesize that this contributes to these results. Also, as expected, our ongoing experience with four-tract tractography has further decreased the total number of sonications over time, with more recent patients receiving fewer sonications than previously treated patients. Using our methodology, we appreciate some degree of immediate tremor response and an absence of side effects at the first target. Also interesting but unexpected was the decrease in the threshold of the thermal dose required for the initial tremor response. Previous papers have indicated that tremor response can be detected with a threshold of 51–54°C.^1^ We observed tremor response at an average temperature of 47.5°C. This observation will require a prospective study. Our practice today is to definitively treat after the alignment sonications show an improvement in tremor without the development of adverse effects. This represents an evolution of our trust in our tractography method.

The Archimedes Spiral has been shown to highly correlate with overall tremor score as measured by standardized tremor scales.^23^ To objectively quantify overall tremor severity before and after MRgHIFU we utilized the Archimedes spiral and demonstrated an overall average tremor reduction of 81% at 3 months. Although it is difficult to compare to prior studies that utilized various tremor scales, Lak *et al.*^7^ reported an 86.2% reduction in extremity tremor at 3 months using the Fahn–Tolosa–Marin rating scale of the treated limb. In our study, an unexpected clinical outcome was the abatement of axial tremor and voice tremor in 12 patients. Previous work has identified the roles of the primary somatosensory cortex and SMC in maintaining axial posture and voice control, especially in the movement preparation phase.^33,34^ We hypothesize that axial and voice tremor improvement occurred from targeting the confluence of the dDRTT and ndDRTT tracts, causing ablation of the tracts projecting to the supplementary motor and premotor cortices. Our experience suggests that the proximity of the FUS lesion to the ndDRTT is important for extremity tremor control and the proximity of the FUS lesion to the dDRTT is important for axial and voice tremor control.

There are several limitations to this manuscript. First, the crossing fibres of the dDRTT can be challenging to reproduce accurately. However, modification of the red nucleus ROI and adjusting FA parameters can improve the performance of fibre tracking algorithms and then identifying fibre bundles based on anatomic knowledge improves accurate reproducibility of these tracts. Second, there is a lack of tremor scale assessments in this study. While many historical papers utilize a variety of tremor scales to score tremors before and after treatment, we chose to use the Archimedes spiral test. While the Archimedes spiral has been validated as a screening tool for ET and also highly correlates with overall tremor score, it lacks patient-reported metrics identified in many standardized tremor scales.^23^ Third, we report patient outcomes at 3 months. Although this is an established time point to gauge response to therapy as oedema has resolved; prior studies suggest that there is some tremor recurrence at 12 months. While other groups have reported an improvement in adverse effects at 12 months, the only adverse effect we encountered was a transient subjective imbalance, which resolved 1 month after treatment. Fourth, this technical note is a retrospective study design. Although prospective, randomized, double-blinded studies are better study designs, we do not consider randomization of patients for this purpose to be appropriate given our experience and results. Primarily, the increased risk of side effects associated with indirect targeting. There are also some limitations to adapting this method widely, especially at sites with limited access to MRI and limited tractography expertise.

Advances in neuroimaging are improving the ability to target white matter tracts. This report provides an approach for four-tract tractography using FDA-approved software for use with MRgHIFU and DBS. Our experience suggests that this is a reliable and seemingly precise method to achieve tremor control. The method accounts for individual variability rather than relying on landmark-based (indirect) targeting, which, although standardized, remains imperfect. Further multicenter prospective validation and formal comparative evaluation are warranted to further assess the reproducibility, clinical effectiveness and safety of this approach because preliminary open-label analysis suggests promise.

## Supplementary Material

fcac273_Supplementary_Data

## Data Availability

The datasets that support the findings of this report are available on request from the corresponding author. The data are not publicly available due to information that could compromise the privacy of participants.

## Abbreviations

AC =: anterior commissure
ET =: essential tremor
MRgHIFU =: magnetic resonance–guided high intensity focused ultrasound
PC =: posterior commissure
SDR =: skull density ratio
VIM =: ventral intermediate nucleus
