# Supplementary material for: Focused ultrasound using a novel targeting method four-tract tractography for magnetic resonance–guided high-intensity focused ultrasound targeting
Source: Brain Commun. 2022 Oct 25;4(6):fcac273. doi: 10.1093/braincomms/fcac273 (PMC9897190; doi:10.1093/braincomms/fcac273)
Supplement: fcac273_Supplementary_Data [file fcac273_Supplementary_Data.docx]

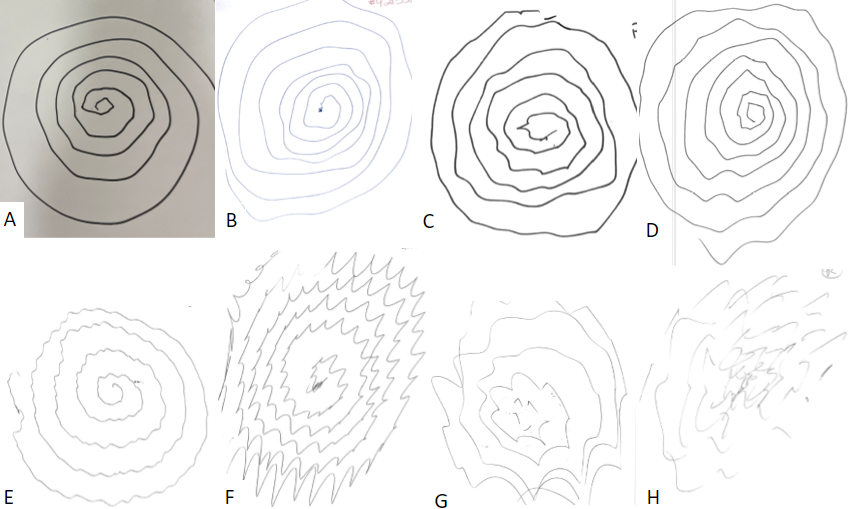


**Supplementary Figure 1**: Examples of spiral scores rated with a modified WHIGET Tremor Rating Scale. **(A)** Grade 0.5; **(B)** Grade 1; **(C)** Grade 1.5; **(D)** Grade 2; **(E)** Grade 2.5; **(F)**. Grade 3; **G**. Grade 3.5; **(H)**. Grade 4.

**Supplementary Table 1: Summary of Treatment Parameters**

| **Average SDR** | **0.50 ± 0.09** |
| --- | --- |
| **Total Number of Sonications** | **9.9 ± 1.7** |
| **Total Number of Treatment Sonications (Post-Alignment)** | **5.2 ± 1.2** |
| **Maximum Average Temperature (Celsius)** | **57.5 ± 1.7°C** |
